# Seluge++: A Secure Over-the-Air Programming Scheme in Wireless Sensor Networks

**DOI:** 10.3390/s140305004

**Published:** 2014-03-11

**Authors:** Farzan Doroodgar, Mohammad Abdur Razzaque, Ismail Fauzi Isnin

**Affiliations:** Faculty of Computing, Universiti Teknologi Malaysia, Skudai, 81310 JB, Malaysia; E-Mails: farzan.doroodgar@gmail.com (F.D.); fauzi@cs.utm.my (I.F.I.)

**Keywords:** wireless sensor networks, Seluge, Merkle tree, over-the-air programming, security

## Abstract

Over-the-air dissemination of code updates in wireless sensor networks have been researchers' point of interest in the last few years, and, more importantly, security challenges toward the remote propagation of code updating have occupied the majority of efforts in this context. Many security models have been proposed to establish a balance between the energy consumption and security strength, having their concentration on the constrained nature of wireless sensor network (WSN) nodes. For authentication purposes, most of them have used a Merkle hash tree to avoid using multiple public cryptography operations. These models mostly have assumed an environment in which security has to be at a standard level. Therefore, they have not investigated the tree structure for mission-critical situations in which security has to be at the maximum possible level (e.g., military applications, healthcare). Considering this, we investigate existing security models used in over-the-air dissemination of code updates for possible vulnerabilities, and then, we provide a set of countermeasures, correspondingly named Security Model Requirements. Based on the investigation, we concentrate on Seluge, one of the existing over-the-air programming schemes, and we propose an improved version of it, named Seluge++, which complies with the Security Model Requirements and replaces the use of the inefficient Merkle tree with a novel method. Analytical and simulation results show the improvements in Seluge++ compared to Seluge.

## 1. Introduction

Wireless sensor networks (WSNs) are used in many areas, like military zones [1], industry automation, vehicular *ad hoc* networks [2] and healthcare systems [3], *etc.*, where security is a very important issue [4]. Furthermore, the wireless medium used as the communication channel in WSN is considered to be an insecure and untrusted way of communication in which an attacker can easily eavesdrop, inject, delay, modify or remove any packet transmitted through a network [5]. In particular, the use of wireless channels as the communicating medium in network protocols makes such a network vulnerable to different types of attacks [6].

WSNs are comprised of numerous physical nodes, and each of these has a programmable microcontroller. The operational behavior of WSNs will be implemented in the Electrically Erasable Programmable Read-Only Memory (EEPROM) of these microcontrollers. The code programmed in the nodes will finally shape the network. For instance, since a routing protocol is required for WSNs to form the topology of the network, this protocol will be coded and compiled into a binary image file. Then, this binary file can be written into EEPROM for execution throughout the network. Installing programs into nodes is possible either by plugging each node into a PC and using external programming devices or by pushing updates into nodes remotely using a base station, which is generally a more powerful system compared to sensor nodes. The first approach is not very reasonable, due to the large number of nodes in WSNs. Hence, the over-the-air programming (OTAP) of the code updates in wireless sensor networks have been researchers' point of interest in the past few years [1,6–15]

Many OTAP schemes or protocols have been proposed (e.g., Deluge [7], Multihop Network Programming Service (MNP) [8], Multihop Over-the-Air Programming (MOAP) [9], Aqueduct [10]), and most assume non-malicious nodes. This assumption is inadequate in many applications, since for these applications, we need to maintain four security aspects (confidentiality, integrity, authentication and availability) in OTAP protocols [16]. Therefore, several vulnerabilities exist when an OTAP protocol wants to propagate an update code remotely. An eavesdropper can threaten *Confidentiality* by gaining information through sniffing image data being disseminated to nodes [17]. The *Availability* of the network is threatened, because the adversary may inject bogus packets during the code propagation to force sensor nodes to propagate the corrupted image, potentially over multiple hops, to deplete their limited power [18]. An adversary can inject a malicious update into the system. Given the epidemic nature of network reprogramming protocols, an adversary can gain complete control over the entire sensor network by compromising just a single node [11]; therefore, the *Authenticity* of the network is threatened. *Integrity* can also be threatened if an adversary modifies update packets before they arrive at the receiver end. Additionally, replay attacks, battery-drain attacks and wormhole attacks are also possible on OTAP protocols and their existing proposed security models [1,6,11–15].

Given the situation, security needs to be supported in any serious reprogramming system [16]. The security attributes for security sensitive applications in *ad hoc* networks (authentication, integrity, confidentiality and availability) are well defined [19] and apply to WSN, as well. The challenge to achieve this goal is the severe resource constraints of WSNs, namely the limited memory, energy, bandwidth and processing power. Therefore, the overhead of a secure OTAP scheme has to be minimum compared to the security strength achieved.

Considering the above issues, in this work, we analyze the existing security models used in over-the-air dissemination of code updates for possible vulnerabilities, and then, we provide a set of countermeasures, correspondingly named Security Model Requirements (SMR). Based on the analysis, we concentrate on Seluge [12]. Security-wise, it is one of the most promising OTAP protocols. It uses the Merkle tree structure. Including the inefficiency of the Merkle tree, it suffers, due to: (i) an exponential growth in the number of overhead packets when a block size of the hash algorithm used in the design is increased; and (ii) the limitation of the hash algorithms with a larger block size. To handle these issues, we propose a new OTAP protocol named Seluge++, which complies with SMR and uses an efficient method compared to the Merkle tree. Analytical and simulation results show the potential of Seluge++ compared to Seluge.

Previous efforts on securing the dissemination of code updates in WSNs are studied in Section 2. Section 3 analyzes the weaknesses of Merkle trees to show that previous works that have employed the Merkle tree in their design are vulnerable to attacks. Assumptions and threat model are presented in Section 4. Afterward, in Section 5, a list of prerequisites required in the design of a secure OTAP scheme is identified, which is correspondingly named the *Security Model Requirements (SMR)*. The design details of Seluge++ that address the current issues with OTAP schemes in scenarios where security must be at the highest level possible are given in Section 6. The behavior and application of the message-specific puzzle as a weak authentication mechanism used in Seluge++ is described in Section 7. The results of security analysis and performance evaluation in Section 8 show Seluge++ establishing a balance between energy consumption and the provided security level. The compatibility of Seluge++ with SMR will also be presented in this section. Finally, the conclusion comes in Section 9 to sum up our findings.

## 2. Related Work

Several works have been proposed recently for secure code dissemination in wireless sensor networks (WSNs) [11,12,20–22], and most of these are extensions to Deluge [7]. Deluge is a code dissemination protocol that is insecure. The updating data in its underlying protocol is divided into a number of pages, and each page consists of a constant number of packets. Security models based on Deluge usually exploit the property of Deluge that causes nodes not to accept packets from any page unless all the packets of the previous page are received.

There are two opposing elements in designing secure OTAP schemes: a complete solution for providing security and resource consumption. All the research that has been carried out in this context is trying to create a reasonable balance between these two. However, existing OTAP schemes either have left behind a key principle of security or have accepted an unreasonable overhead, incurred by their protocols.

The authors of Secure Deluge [23] have proposed including the hash value of each packet into its previous packet, and then, the hash value of the first packet will be included in an advertisement packet, which is signed with the private key of base station. The downside is that when a packet arrives out of order due to the multi-hop nature of WSNs, it cannot be authenticated unless all the previous packets are received. Although they have suggested a technique of storing out of order packets in a buffer, in turn, this will open a backdoor for intruders to exploit the possibility of buffer overflow and, consequently, the possibility of performing a successful denial of service (DoS) attack.

Munivel *et al.* have proposed the *μ*PKI (Micro Public Key Infrastructure) scheme [21]. This protocol takes the benefits of public key cryptography in combination with symmetric cryptography and covers the issues regarding the storing of the symmetric key in nodes. It generally consists of two handshakes, which shapes the infrastructure of this scheme. One is the node-to-node handshake, and the other one is the base station-to-node handshake. The main disadvantage of this scheme is the initial overhead caused by the creation of a temporary shared key using PKI to be used for a symmetric cryptosystem. The public key of the base station will help to preserve the authenticity, and by a message authentication code (MAC), attached to every packet, the integrity of the updates will be enforced. Finally, for privacy preservation, a temporary shared key, which will be updated periodically, will be used. However, referring to the statements of the author, the downside is that *μ*PKI is not protected against DoS attacks.

The Merkle hash tree was first used in [22] for a hybrid scheme, which sets the number of required public cryptography operations to a single signature-verification, so that in each page, they construct a Merkle tree of hash values of packets in that particular page. Although this method provides an authentication and integrity check, it has a high overhead, because of the need to send extra bytes of the Merkle tree for every page. This protocol also does not provide confidentiality preservation. Furthermore, the transmission of packets in the Merkle tree is level-based, meaning that packets of level 3 cannot be verified unless all the packets in level 2 are delivered. This behavior causes a hybrid scheme to be vulnerable to DoS attacks.

Caloca de la Parra *et al.* proposed to modify the Merkle tree structure, so that the number of packets in each page will be 15 bytes [16], which will result in an inefficient dissemination protocol. In their scheme, for each page, a new Merkle tree needs to be created, and then, using nodes in this tree, four hash packets will be constructed. These packets will be sent in the beginning of the transmission of each page before the transmission of data packets starts. Apart from the high overhead incurred being the same as the hybrid scheme, due to the use of Merkle tree nodes per page, it is also vulnerable to DoS attack, as an intruder can easily attack the dissemination protocol by halting the transmission of hash packets, which are sent *a priori*.

**Figure 1.**
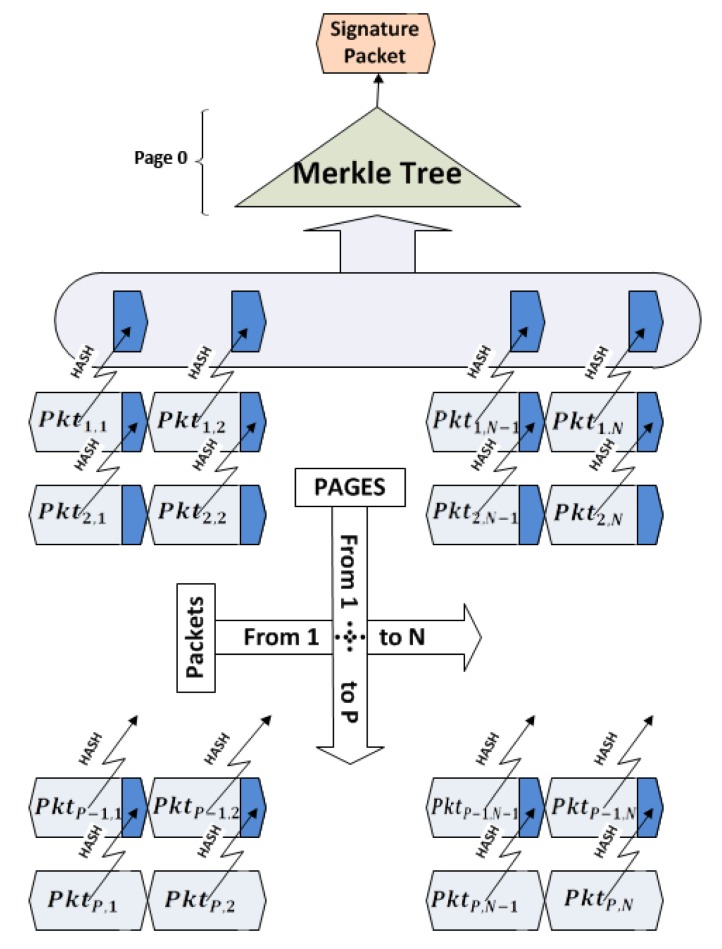
Authentication and integrity check of the code image in Seluge (adapted from [12]).

**Figure 2.**
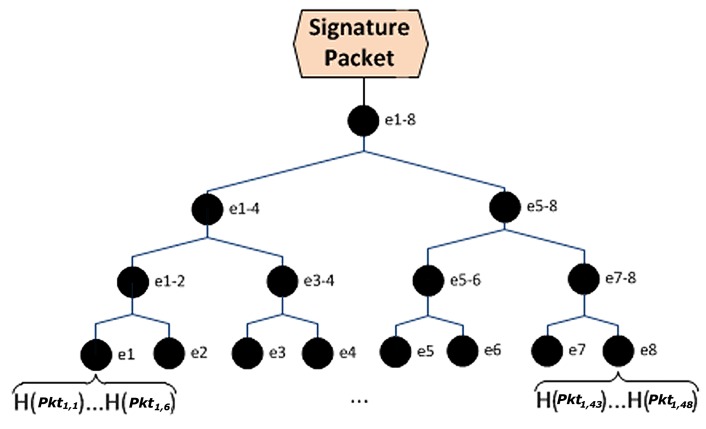
The structure of the Merkle hash tree used in Seluge (adapted from [12]).

Hyun *et al.* have proposed Seluge [12], where they have used a new, efficient pattern for constructing hash values out of the data packets. Figure 1 illustrates their protocol and describes the pattern this scheme uses. The hash value of each packet in page P is inserted to its corresponding packet in the previous page (P-1). This procedure takes place, until all the hash values of the packets from the last page to the first page are created and placed in their relative packets. Seluge proposes to attach all the hash values of page 1 sequentially to create *HashValues* = *H*(*Pkt*_1,1_) ⋯ *H*(*Pkt*_1_,*_N_*), and then, these values will be split into *M* = 2*^k^* fragments, where K will be the minimum value that fits in Equation (1). Note that resulting M is the number of leaf nodes of the Merkle hash tree (Figure 2). |*H*(.)| is the block length of the hash algorithm in bytes. For example, 128-bit MD5will be denoted as |*H*(.)| = 16, and N is the number of packets in each page.

(1) $$\frac{\left(N\times |H(.)|\right)}{2^{K}}+K\times |H(.)|\leq \text{MaximumPayloadSize}$$

Note that maximum payload size in IEEE 802.15.4 compliant radios is defined to be 102 bytes per packet.

Seluge also suffers from a security limitation and a high overhead, which will be discussed later in Sections 3 and 8.

Existing security models for OTAP schemes are summarized in Table 1. Note that Deluge in the first row of Table 1 is not a security model and is the basic infrastructure used in all the discussed security models.

The authors of Deluge have not considered any security principles while designing Deluge; therefore, this OTAP scheme has posed high security risks and is vulnerable to all types of attacks. This fact in Table 1 is shown by adding a cross for all cells of Deluge's row. All other rows in Table 1 are assigned to one security model on top of Deluge that is partially preserving the security principles and that is resilient to a few attack types. *μ*PKI preserves confidentiality, integrity and authentication while all other models preserve integrity and authentication; however, they miss the privacy concerns among them that Seluge is resilient to denial of service, wormhole and replay attacks, while others are vulnerable to many attack types. Meanwhile, we can see that each model that has tried to make a more secure scheme is suffering from a very high overhead. The Hash tree scheme suffers from very high overhead, while comparing this scheme with its equivalents in terms of being secure, Sluice and Das and Joshi, which have less overhead. This implies that the authors usually could not keep a balance between the security strengthening that their scheme provided and the overhead that was incurred in their scheme.

Comparing Seluge with other schemes listed in Table 1, there is less work involved to make it more secure, because it is already robust against many attacks, while others are not. Seluge does not preserve Confidentiality and is not robust against battery-drain attacks. However, its Dos attack resilience is acceptable in most in-field usage, but it is not enough to be used for mission-critical purposes. Seluge's overhead is also very high and needs to be reduced. Having said all that, Seluge is chosen to improve its attack resilience, performance and overhead, while keeping the balance between these.

## 3. The Limitations of Seluge's Merkle Tree

Referring to Equation (1) defined in Section 2, in Seluge, to identify a value of *M*, we have to fill the equation with the default values of *N* = 48, |*H*(.)| = 8 and *payload size* = 72. Authors of Seluge have used a truncated 64-bit SHA-1, which is more vulnerable to collision attacks compared to 160-bit SHA-1. However using Seluge's default values, the value of *M* will be equal to eight (k = 3).

Unfortunately, for situations where security must be at the highest possible level (e.g., battlefields, healthcare systems), using 64-bit SHA-1 is not recommended. Furthermore, as shown below, Seluge cannot afford to use 160-bit SHA-1, because no value of integer number (*K*) will satisfy Equation (1) when |*H*(.)| = 20(160 *bit*). This statement remains true, even if we use a maximum payload size instead of Seluge's default value of 72:

$$\frac{\left(48*20\right)}{2^{K}}+K*20⪯102$$

The only solution for this problem is to decrease Seluge's default value of the number of packets per page from *N* = 48 to a possible maximum of *N* = 17, which then will result in the *K* to satisfy Equation (1) with a value of four, consequently creating a Merkle hash tree with 2^4^ = 16 leaf nodes. This value for *N* is an awkward selection, because it will highly degrade the performance of Deluge as the underlying dissemination protocol.

Our findings show that this Merkle hash tree is not highly secure and efficient enough to be used in situations where security is critical, and therefore, usage of this tree-based mechanism is not suggested in mission-critical situations like military zones or generally in networks that are related to human safety. However, the way that Seluge has employed this method still provides a reasonable security level compared to other similar mechanisms [16,25] for situations where security does not need to be very strict.

Later, in Section 8, we will show how much overhead this structure of the Merkle tree incurs in the dissemination of the code updates in Seluge.

## 4. Assumption and Threat Model

**Assumptions:** The network is assumed to be deployed in a situation where all the security principles, like privacy, confidentiality, integrity and availability must be preserved (e.g. military zones). The network is vulnerable to DoS, replay, wormhole and battery-drain attacks. Nodes are assumed to be computationally constraints and are not tamper-proof devices. A node is therefore assumed to be able to perform a limited number of public cryptography operations. A base station (BS) with powerful computational capabilities (e.g., a laptop) with no energy consumption limitation is assumed and a private/public key pair is associated with this BS. All the sensor nodes are then preconfigured with a public key of a BS before the deployment of the network. Deluge is assumed as the underlying over-the-air programming (OTAP) protocol.

**Threat Model:** An attacker has a powerful set of devices, both in terms of computation and communication medium. As node communications are based on a wireless medium, it is assumed that there is an untrusted and insecure channel in which an attacker can eavesdrop, inject, delay, modify or remove any packet transmitted. The base station cannot be compromised by attackers; therefore, the public/private key pair is always safe. Jamming attacks are possible on the network, and since a jamming attack is out of the scope of this study, it is assumed that the adversary cannot perform a permanent jamming without being detected and removed. The attacker might try to disseminate malicious code updates into a network or also replay previously sent packets (replay attack). The attacker also might perform denial of service (DoS) attack on the network.

## 5. Security Model Requirement

As concluded in Section 2, although some good achievements have been obtained in terms of integrity and authentication, existing security models for code updates in WSNs are lacking some critical requirements, like availability or confidentiality. As a result of studying all these protocols, we have identified a list of security considerations (Table 2) in designing a secure protocol. In the case of an over-the-air programming scheme for WSNs, it is necessary to make sure that it is robust against all possible attacks and that all the security principles are preserved. It is mandatory to implement these prerequisites in the design of the OTAP scheme. A review on how this model secures OTAP schemes can be found below:

**Confidentiality:** For confidentiality preservation, symmetric cryptography (e.g., AES) has very less energy consumption compared to public key cryptography algorithms [26] (e.g., RSA). Furthermore, storing a symmetric key on each node is not a wise idea, because an attacker can easily obtain it in the case of a non-tamper-proof device. Therefore, for confidentiality preservation, it is suggested to use symmetric cryptography. A symmetric key needs to be avoided from being written on the EEPROM of nodes for further use, and instead, this key has to be unique and new for each communication link and must be generated so that it is authentic. For the sake of simplicity, we refer to this temporary key as the session key. In our protocol, the elliptic curve Diffie–Hellman (ECDH) key exchange method [27] is used, and the authenticity of the generated session key is guaranteed using signature verification. Communication privacy can also be provided using a link-layer encryption system, like TinySec [23,28].

**Authentication:** A secure scheme needs to be compatible with all types of public key cryptography (e.g., RSA, elliptic curve cryptography (ECC)), and depending on the network situation, different algorithms can be employed. In the TinyECC library [26,29], elliptic curve cryptography shows better performance over RSA (Table 3).

## 6. Design of Seluge++

**Figure 3.**
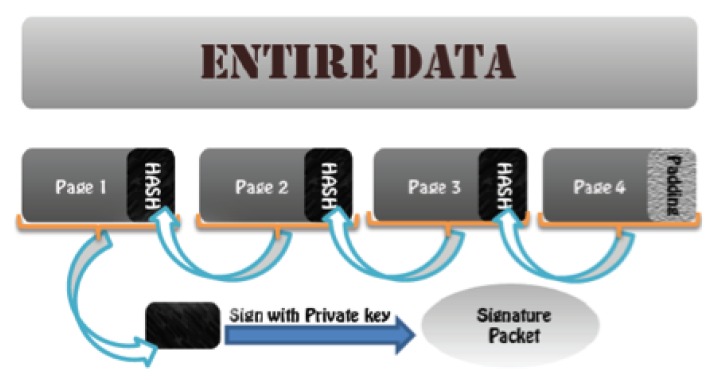
Integrity verification using hash dependency.

**Integrity:** The integrity check mechanism using hash values with the help of signature verification can guarantee that all the data are coming from a trusted source and that no modification is made on them by a malicious node. WSNs are limited in the number of public cryptography operations that they can perform in their lifetime; hence, we cannot sign every packet for the sake of integrity verification. Therefore, algorithms, like Hash-Chain, Hybrid and Sluice, have been proposed that use single public key signature verification with the use of a hash dependency list or tree. The general idea of using hash dependency and signature verification for integrity verification is shown in Figure 3. If symmetric cryptography is used, message authentication code is also another approach, which is applicable in WSNs for integrity purposes.

**Figure 4.**
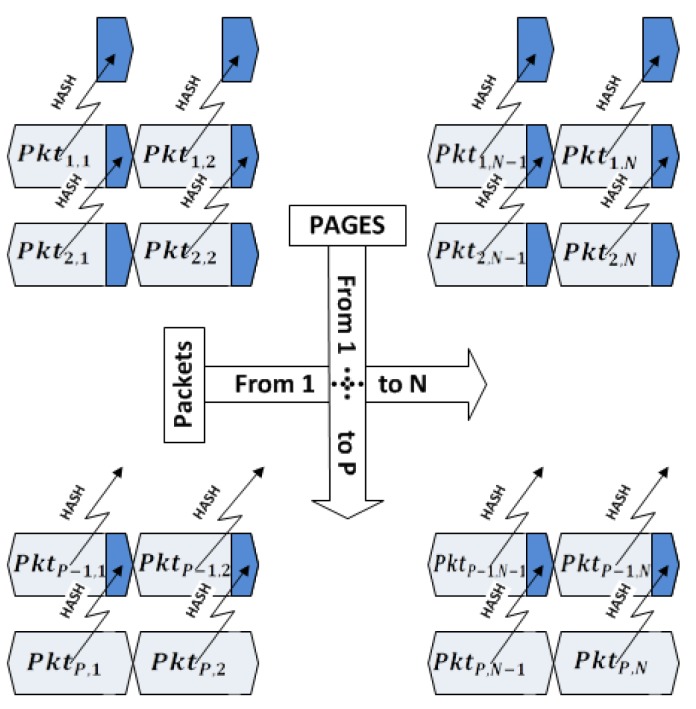
The hash dependency structure in Seluge (adapted from [12]).

However, studies performed by the authors of [12,16,22,23] show that a successful way for authenticating image code has to be at the packet level. This requires assigning hash value for each packet in previous packets using a pre-defined pattern, so that each packet being received in the nodes can be immediately verified by its previously received hash value. The most efficient pattern to the best of our knowledge is employed in Seluge (Figure 4). Therefore, SMR suggests using this pattern for the hash dependency algorithm.

**Mitigating DoS Attack:** DoS attack resiliency is an important factor, which has either been neglected or partially preserved in the design of all the security models of WSN reprogramming protocols. Although hash dependency is provides a good and efficient way of authenticating data and preserving integrity, it is susceptible to DoS attacks. Moreover, out of order packet delivery is a regular occurrence, because of the collision in typical wireless networks, and it can cause data in page P to arrive before page P-1, therefore causing the receiving node to buffer these packets, as it cannot verify it unless all the packets in page P-1 are received. This problem is also known as *authentication delay*. If an attacker sends a lot of bogus data or packets indicating that they are from higher numbered pages, then the node will need to either buffer them all or simply drop them. None of these approaches is efficient enough to be employed in WSNs, since, by nature, this incident can be due to network collisions, which are common in some scenarios, and therefore, the node will consume a lot of energy sending data that will be dropped by other nodes or that will be buffered. This behavior results in a buffer overflow and, consequently, a successful DoS attack. In the practical usage of data authentication, previous works have shown that packet-level verification provides more powerful protection against DoS attacks [23]. Consequently, to have a reasonable DOS resiliency, an *immediate authentication per packet* needs to be employed. Thus, we propose to use a *combination of hash dependency* and *message authentication code (MAC)* in SMR. Therefore, the out of order delivery of packets that causes *authentication delay* problem is solved, and an immediate authentication per packet is achieved, while the integrity of the data is still preserved. This combination will help to preserve the integrity and authenticity and mitigate DoS-attacks. This combination will be described in detail in Section 6. Hash dependency has been proposed in different forms by many authors [11,22,23], but not all of them provide an acceptable balance between DoS resiliency and integrity check strengthening. However, the pattern employed by the authors of Seluge to construct hash values has a reasonable balance in scenarios where the requested security level is up to a standard level, but not when the security level needs to be at a maximum level.

**Mitigating Replay Attack:** Replay attacks are very easy to practice against the reprogramming of wireless sensor networks, due to the use of broadcast mechanisms in transmitting update data. To mitigate this type of attack, we need to define a property named freshness. This means that update data that have previously been activated on nodes will not be accepted for installation on nodes, even though it is authenticated data. We use a version counter that is the same as in [15] to merge this property into SMR.

**Figure 5.**
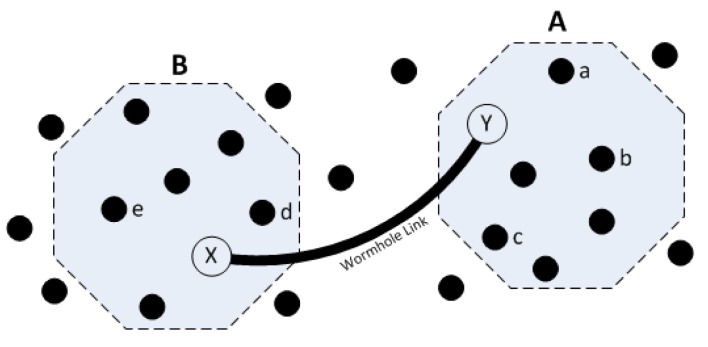
Wormhole attack in a wireless sensor network (WSN) [6].

**Mitigating Wormhole Attack:** Wormhole attack can be practiced against the reprogramming of WSNs [6,14] if a symmetric key is used throughout the whole network, giving enough time for the attacker to find out the symmetric key using cryptanalysis and then sending forged packets in parts of the network that an authentic code update has not yet been disseminated. Figure 5 illustrates a wormhole attack on WSNs. The attacker creates a fast link between areas A and B of the network. Assume that a dissemination of the code update is started by node *a*, and data has reached all the nodes in area A, but it will take time to reach area B. Meanwhile, the attacker will sniff the data transmitted in A, and if she can exploit the symmetric encryption used in the running protocol and extract the shared key, she can use this key to exploit all the nodes in B. As mentioned earlier, we will use a unique and temporary session key per communication link; therefore, this attack cannot be practiced against schemes that comply with SMR.

**Mitigating Battery-Drain Attack:** Battery-Drain attack is very common against authentication packets in which the attacker will send bogus authentication packets. This forces the receiver to perform a verification process, which is very power consuming in WSNs and leads to battery-drainage. NACK packets introduced in Deluge are also susceptible to battery-drain attacks. The solution to this problem is the use of a weak authentication. The selected method for this purpose has to be very easy for the node to generate, but computationally infeasible for adversaries to calculate the inverse. The most well-known methods that have this property are hash algorithms. A message-specific puzzle, proposed by [30], effectively takes the benefit of the one-way functionality of hash algorithms and can mitigate battery-drain attacks and also DoS-attacks that are against authentication and NACK packets.

**Figure 6.**
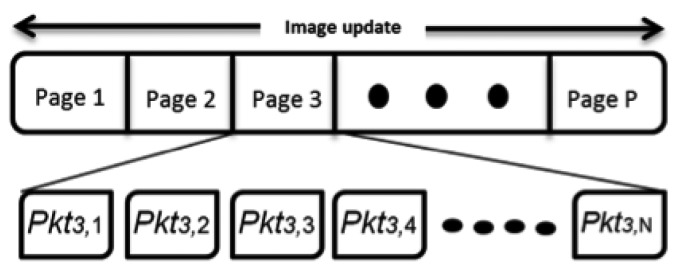
Seluge++ code partitioning.

As previously highlighted, Seluge++ is an extension to Deluge; therefore, it conforms to the basics of the Deluge protocol. The same as Deluge, an update image will be divided into equal pages denoted as page 1 through page P, except the last page, which might be smaller; therefore, it will be filled with padding bits to fulfill this requirement. Each page, *i*(1 ≤ *I* ≤ *P*), will have a default number of packets, *N*, denoted as *Pkt_i_*_,1_ through *Pkt_i_*_,_*_N_* (Figure 6). By default, *N* = 48.

### 6.1. Immediate Packet Verification

To achieve the desired compatibility with SMR, similar to Seluge, we reserve some bytes of each packet for assigning hash values. The length of these values differs depending on the hash algorithm used. In Seluge++, to provide the highest security, we use 160-bit SHA-1, which will need 20 bytes per packet. We will also consider 72 bytes of update data to be stored in each packet; therefore, a sum of 92 bytes will be used as the default payload, which is less than the maximum of 102 bytes defined in IEEE 802.15.4. Like the original Seluge, the hash value of each packet in last page P is inserted to its corresponding packet in the previous page (P-1). This procedure takes place until all the hash values of the packets from the last page through the first page are hashed and placed in their relative packet. For example, assume there are 10 pages to be transmitted; therefore, *P* = 10. The base station will start from the first packet in the tenth page (*Pkt*_10,1_) and will store its hash value into *Pkt*_9,1_, continuing this process with storing the hash value of *Pkt*_10,2_ in *Pkt*_9,2_, and so on. The hash values of page 1, which do not have any corresponding packet for storage, will then be called page 0. For the sake of simplicity, this packet preparation process hereafter will be named the Data Verification Method (DV Method), which is already illustrated in Figure 4.

The DV method will only be effective on the verification of data from page 1 through P, but packets in page 0 must also be disseminated in a way that they can be immediately verified. In Section 3, we have shown that using the Merkle hash tree does not comply with our assumption of a mission-critical network; therefore, unlike Seluge, we introduce a new method for the verification of packets in page 0. This method benefits from the use of a message authentication code. Since using MAC needs to have a symmetric encryption, therefore Seluge++ will use both asymmetric cryptography for authentication preservation of the entire image data and symmetric cryptography for both integrity preservation and packet verification (using MAC) of hash packets in page 0. The detailed process of this verification will be discussed in the following. Hereafter, for the sake of simplicity, we will recall this verification of packets in page 0 as the *Hash Verification Method* (HV Method).

Seluge++ uses symmetric key similar to *μ*PKI, but better in performance, since the receiver nodes do not need to sign data using public cryptography, whereas in *μ*PKI, they do need to do this. This approach also removes the overhead of the Merkle hash tree.

Therefore, using a combination of the Hash Dependency Method (the DV Method) and message authentication code (the HV Method), the authenticity of updates and also their integrity against any possible modification by attackers is guaranteed.

### 6.2. Key Agreement

As mentioned earlier, Seluge++ will use a symmetric encryption along with asymmetric authentication. Since we assume a non-tamper-proof device and also, in SMR, we highlighted the importance of having a temporary session key per communication link, therefore a key exchange mechanism has to be chosen to securely exchange a symmetric key in the beginning of the propagation phase. As we need to make sure this key generation is triggered by an authenticated source, the key exchange algorithm must be integrated into the asymmetric signature verification process. The best method that suits these conditions and also has an acceptable security level is the elliptic curve Diffie–Hellman (ECDH) key agreement algorithm (Algorithm 1).

As described in RFC-5349 [31], when securing an 80-bit symmetric key, it is prudent to use 160-bit elliptic curve cryptography (ECC). For domain parameters, group P-192 (secp192r1) is suggested to be employed in Seluge++.



**Algorithm 1** Elliptic curve Diffie-Hellman (ECDH) key agreement.
1:Agree upon domain parameters (*p, a, b, G, n, h*)2:**Alice:**3:Private key: *d_A_* ⇐ *A random integer*
**in range:** [1, *n* − 1]4:Public key: *Q_A_* ⇐ *d_A_G*5:Alice's key pair will be (*d_A_, Q_A_*)6:Send *Q_A_* to Bob7:**Bob:**8:Private key: *d_B_* ⇐ *A random integer*
**in range:** [1, *n* − 1]9:Public key: *Q_B_* ⇐ *d_B_G*10:Bob's key pair will be (*d_B_, Q_B_*)11:Compute (*x_k_, y_k_*) ⇐ *d_B_Q_A_*, **where**
*x_k_* is the shared key12:Send *Q_B_* to Alice13:**Alice:**14:Compute (*x_k_, y_k_*) ⇐ *d_A_Q_B_*, **where**
*x_k_* is the shared key


### 6.3. Notations

Table 4 contains the notations that will be used in the remainder of this study.

(2) $$I=\left\lceil \frac{\text{NPPP}\times |H(.)|}{B-|MAC_{SK}(.)|}\right\rceil$$

For Instance, having *NPPP* = 48 which is the default value in Deluge, *B* = 92, which is set to be the Seluge++ default payload size (discussed in Section 6.1) and |*H*(.)| = |*MAC_SK_*(.)| = 20 bytes, if 160-bit SHA-1 is used for high-level security; a value of *I* will be populated as):

$$I=\left\lceil \frac{48\times 20}{92-20}\right\rceil =\left\lceil 13.333∼\right\rceil =14\;\text{packets}$$

### 6.4. Transmission Process

After a code update is available, the base station (server) will start to send advertisement packets until a node requests the update code. Since the session key is needed prior to the propagation phase in order to encrypt data before the hash calculation of packets in the Data Verification Method, the ECDH key agreement process will be the next step that the node and BS will take. The node, after receiving an advertisement packet, will choose its sender and ask for a signature packet. Then, the BS will load *T* (elliptic curve domain parameters) and will select a random number 1 < *d_S_* < *n* − 1 and a random 160-bit number (*N*_0_); then, the BS will calculate *Q_S_* = *d_S_G*. The server will sign a packet containing *V_n_* (to keep freshness), *I, N*_0_ and *Q_S_* with his *GK_Pri_* and will send this to the node.

$${\text{Sign}}_{GK_{\text{Pri}}}(\{V_{n},I,N_{0},Q_{S}\})$$

The node will receive that packet and will decrypt its content using *GK_pub_*, which is configured in nodes *a priori*. The value of *I, N*_0_ and *Q_S_* will temporarily be kept in the RAM storage of node. Before the node takes any other action, it will compare the value of *V_n_* with the value in the earlier disseminated code; if the new value is less or equal to the old value, this means that a reply attack is ongoing. The node will stop the current process and will notify the BS about this attack and will also halt the Seluge++ mechanism for a pre-configured amount of time to prevent resource exhaustion. Otherwise, if the new value of *V_n_* is greater than the old value, the node will continue. In this phase, the node needs to do the rest of the ECDH key agreement procedure by selecting a random number of 1 < *d_N_* < *n* − 1, calculate *Q_N_* = *d_N_G, SK* = *H*^80^(*d_N_Q_S_*) and a random 160-bit number (*N*_1_), where *SK* will behave as the session key mentioned in Section 5. In this stage, the node can free the memory used for storing large numbers, like *G* and *P*.

Then, the node will create packet data in the following format and will send it to the BS:

$$\left\{Q_{N},Enc_{SK}^{80}(N_{1}),MAC_{SK}^{160}(Q_{N}⊕N_{0})\right\}$$

Upon receiving the previous packet, the BS will first calculate *SK* = *H*^80^(*d_S_Q_N_*) and then checks if the generated session key (*SK*) is authentic or not using Equation (3). If it is a valid session key, then it will run a calculation (Equation (4)) to extract *N*_1_. If the generated session key is found to be bogus, then it means an attacker is trying to send forged packets to the BS; since the BS is assumed to be powerful, it is very unlikely for an attacker to perform a DoS attack by sending many reply packets from a malicious node.

(3) $$\left\{\underset{⊗N_{0}\Rightarrow MAC_{SK}^{160}(Q_{N}⊕N_{0})\Rightarrow }{\underset{︸}{Q_{N}},Enc_{SK}^{80}(N_{1}),}\underset{\underset{?}{⩵}}{\underset{︸}{MAC_{SK}^{160}(Q_{N}⊕N_{0})}}\right\}$$

(4) $$Dec_{SK}^{80}(Enc_{SK}^{80}(N_{1}))=N_{1}$$

**Figure 7.**
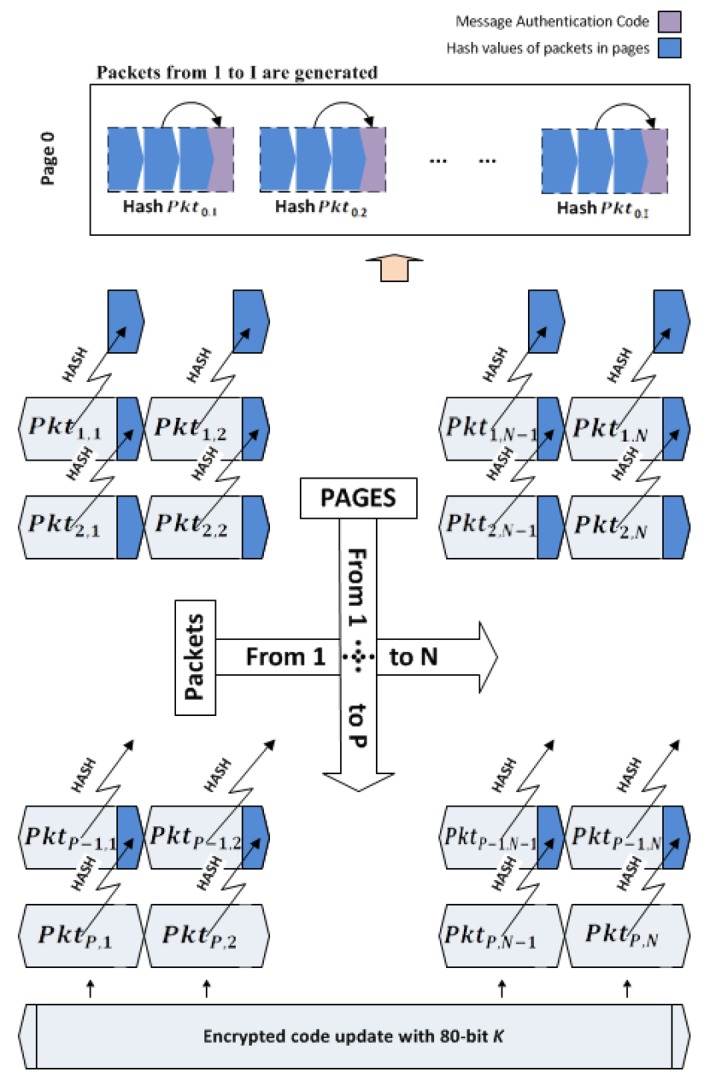
Packet preparation in Seluge++.

Now, the BS has the session key; therefore, it can start the preparation of the code update packets. As specified in the design of Seluge++, the BS will fragment code updates in compliance with Figure 6, and then, the default behavior of the original Seluge will be applied on the generation of hash packets. The key difference is that in the design of Seluge++, to preserve confidentiality, we have proposed to use a temporary session key to encrypt data packets to prevent malicious nodes from understating the behavior of the network by disassembling the code update. In most of the proposed secure dissemination protocols, data encryption has been omitted, because the authors have mostly assumed that in a network, the code update does not need to be protected against a sniffer's willingness to disassemble data. However, our assumption is to have a mission-critical network, like a military zone, in which binary code must be protected, otherwise the attacker can discover the network behavior and, therefore, exploit its applications or try to force the network to generate invalid data. Therefore, prior to the preparation of pages, the entire update image will be encrypted using SkipJack 4 block cipher with an 80-bit key length and 64-bit blocks in CBC (cipher-block chaining) mode, and then, the normal procedure will be started until all the packets in page 0 are generated (Figure 7).

In the next step, the Hash Verification Method (HV Method) will be continued by sending hash packets of page 0 from 1 − *st* through *I* − *th* to the node using the following equation:

$$\left\{Enc_{SK}^{80}(Pkt_{0,i}),MAC_{SK}^{160}(Enc_{SK}^{80}(Pkt_{0,i}))\right\}$$

The node then can verify the integrity of receiving hash values using MAC and the authenticity of them using the fact that the session key used in MAC is generated with the help of the BS signature and the ECDH key agreement technique. Note that upon initialization of this step, the node can free the memory that is reserved for *Q_N_*.

As soon as the transmission of the hash values finishes, the HV Method is completed, and the server will initiate the DV Method to send code updates. By this time, the node already has all the hash values of page 1, which are sent to it in an authentic way. As a result, the hash dependency feature, which is employed in the packet preparation of the code update, can guarantee an immediate verification of every packet in pages 1 to P. This is done by exploiting the fact that the Deluge infrastructure states that a node will neither request the dissemination of page P nor accept packets from page P unless all the packets in page P-1 are successfully delivered and verified. When all the packets are delivered to the node, then the node will free the space reserved by variables, like *I* and also *SK*, after decryption. It will put all the pages together and will decrypt the data file to extract the actual update image. Finally, although replay attack is mitigated using *version number*(*V_n_*) used during dissemination of the code update, for more security, Seluge++ will halt its mechanism for a pre-configured amount of time to prevent attackers from any possible replay attacks after a successful reprogramming of the network. Note that during an ongoing dissemination with an established session, nodes will refuse to accept packets that are previously received and successfully authenticated. This is to make sure that no replay attack can be practiced against Seluge++. The workflow of the dissemination of the code update is shown in Algorithm 2.

## 7. Weak Authentication

As described in Section 5, the message-specific puzzle (MSP) can be used as a weak authentication to prevent nodes from performing complex cryptographic operations when a forged packet arrives. For more details about the behavior of message-specific puzzle, see [30]. Unlike other protocols, in Seluge++, since we are using symmetric cryptography and MAC authentication, it will not be very energy consuming to perform a decryption algorithm compared to asymmetric signature verification. However, we suggest using message-specific puzzles only when needed, in case of attacks taking place in the network. Detection of an attack in which the attacker is sending forged packets is easy; therefore, we will define a threshold after which the node will challenge the server (BS) to send the MSP puzzles, so that, afterward, only packets that have the correct puzzle solutions will be considered as semi-authentic. The authenticity of semi-authentic packets then will be evaluated using MAC authentication.



**Algorithm 2** Dissemination of the code update, including the Data Verification (DV) and Hash Verification (HV) Methods).
1:**Base Station:**2:
$I⇐\left\lceil \frac{\text{NPPP}\times |H(.)|}{B-|{\text{MAC}}_{SK}(.)|}\right\rceil$3:*T* ⇐ (*p, a, b, G, n, h*) *elliptic curve domain parameters*4:*d_S_* ⇐ random integer number (1 < *d_S_* < *n* − 1)5:*N*_0_ ⇐ 160–*bit* random number6:*Q_S_* ⇐ *d_S_G*7:*V_n_* ⇐ *Version number*8:Sign *V_n_, I, N*_0_, *Q_S_* using *GK_Pri_*9:Send *Sign_GK_Pri__*({*V_n_, I, N*_0_*, Q_S_*}) to node10:**Node:**11:(*I, V_n_, N*_0_, *Q_S_*) ⇐ *Verify_GK_pub__* (*Sign_GK_Pri__*({*V_n_, I, N*_0_*, Q_S_*}))12:**if**
*V_n_* ≤ *previous V_n_*
**then**13: Notify BS of an ongoing replay attack.14: Halt dissemination protocol for a pre-configured time.15: Exit16:**end if**17:*previous V_n_* ⇐ *V_n_*18:*d_N_* ⇐ random integer number (1 < *d_N_* < *n* − 1)19:*Q_N_* ⇐ *d_N_G*20:*SK* ⇐ *H*^80^(*d_N_Q_S_*)21:Free memory reserved by *d_N_, Q_S_*22:*N*_1_ ⇐ 160 – *bit random number*23:Execute Algorithm 3 (Input: *N*_1_) {**Optional**}24:Send 
$\left\{Q_{N},Enc_{SK}^{80}(N_{1}),MAC_{SK}^{160}(Q_{N}⊕N_{0})\right\}$ to BS25:**Base Station:**26:
$\text{Pkt}⇐\{Q_{N},Enc_{SK}^{80}(N_{1}),MAC_{SK}^{160}(Q_{N}⊕N_{0})\}$27:*SK* ⇐ *H*^80^(*d_S_Q_N_*)28:*MSG* ⇐ *Pkt*[0] ⨁ *N*_0_ {*Pkt*[0] = *Q_N_*}29:*generated*
$\text{MAC}⇐MAC_{SK}^{160}(\text{MSG})$30:**if**
*generated MAC* <> *Pkt*[2] **then**31: Exit.32:**end if**33:
$N_{1}⇐Dec_{SK}^{80}(Enc_{SK}^{80}(N_{1}))$34:Execute Algorithm 3(Input: *N*_1_) **{Optional}**35:Encrypt update image with *SK*36:Fragment encrypted data as shown in Figure 437:**for all**
*Pkt*_0,_*_i_* in Page0 **do**38: Send 
$\left\{Enc_{SK}^{80}(Pkt_{0,i}),MAC_{SK}^{160}(Enc_{SK}^{80}(Pkt_{0,i}))\right\}$ to the node39:**end for**40:**Node:**41:Free memory reserved by *Q_N_*42:**while** Receives 
$\left\{Enc_{SK}^{80}(Pkt_{0,i}),MAC_{SK}^{160}(Enc_{SK}^{80}(Pkt_{0,i}))\right\}$
**do**43: *new MAC* ⇐ *calculate MAC of encrypted Pkt*_0_*_,i_*44: **if**
*new*
$\text{MAC}<>MAC_{SK}^{160}(Enc_{SK}^{80}(Pkt_{0,i}))$
**then**45:  Drop packet46: **end if**47: 
$Pkt_{0,i}⇐Dec_{SK}^{80}(Enc_{SK}^{80}(Pkt_{0,i}))$48: Store *Pkt*_0,_*_i_*49:**end while**


Consequently, the message-specific puzzle feature will be disabled during the normal state of the network, but in order to use it when needed, we might prefer to generate the hash chain a *priori*. This means that the hash chain must be populated into the BS and the node to be used in the case of any emergency situation. One way of doing so, as proposed in [12], is to store the hash chain in every node before deployment of nodes. This causes vulnerabilities, since most of the hash functions that are suitable to be used in a WSN are vulnerable to collision attacks. Therefore, in the long-time usage of a constant hash chain, the commitment of the chain will be revealed. Seluge++ will generate this key chain in the dissemination of each update. However, only for authentication packets, we suggest using the same approach as [12] suggests to store the offline MSP hash chain during the deployment of the nodes, but with a few changes to make sure that it is less susceptible to collision attacks. The key difference is that, for the offline chain that is used as weak authentication only for authentication packets, as far as we are preserving confidentiality and unauthorized entities are unable to understand the content of the transmitted bytes, we suggest updating the hash chain values each time a new update code is disseminated. Therefore, there will be a periodic change, and this will make it difficult for attackers to perform sequential collision attacks against this weak authentication during the lifetime of the network.

As seen in Algorithm 2, the node will generate *N*_1_ and will send it to the server in a secure manner. Therefore, this value is only shared between BS and the node. Using this number as a commitment, a temporary hash chain with I values will be created using Algorithm 3. Algorithm 2 (lines 22 and 33) are places that Algorithm 3 can effectively generate a hash chain. This dynamically generated chain will be used in the weak authentication of packets in the HV and DV Methods.



**Algorithm 3** Temporary hash chain generation of a message-specific puzzle.
1:Get *N*_1_ from input2:*X*_1_ ⇐ *H*^160^(*N*_1_)3:Store *X* in RAM or EEPROM.4:**for**
*i* = 2 to *I*
**do**5: *X_i_* ⇐ *H*^160^(*X*)6: Store *X_i_* in RAM or EEPROM.7:**end for**


## 8. Analysis

In summary, in terms of security, Seluge++ has made a significant improvement compared to the original Seluge. The general advantages of Seluge++ over the original Seluge in terms of security support are shown in Table 5. As shown in Table 5, confidentiality in Seluge++ is preserved using symmetric encryption, while Seluge does not preserve privacy at all. The method that Seluge is using to prevent DoS attacks is not sufficient for mission-critical situations; therefore, Seluge++ improvements using immediate verification at the packet level makes it robust against DoS attacks. battery-drain attacks are now impossible in Seluge++ by the benefiting of the message-specific puzzle. This is while Seluge is vulnerable to battery-drain attacks. Another improvement in Seluge++ is that it supports any block size for the underlying hash function, which results in having a more secure scheme. Seluge could only have a hash function with a maximum of 11 bytes for the block size, which results in Seluge being vulnerable to collision attacks against its underlying hash function.

To show that Seluge++ is maintaining a reasonable balance between the provided security level and energy consumption and performance, five different analyses have been done on Seluge++. These include *security analysis, compatibility with SMR, performance evaluation, energy cost evaluation* and a measurement to show *overhead improvement* and the provided security strengthening of *supported hash block length*. In the following, we present these analyses.

### 8.1. Security Analysis

#### 8.1.1. Casper/FDR2 Approach

To prove the authentication claims of Seluge++, a security model checker is employed that is similar to [32]. FDR (Failures-Divergence Refinement) is a model checker for state automata based on concurrency theory and on CSP (Communicating Sequential Processes) [32,33], and it is mainly used to check the security properties of systems. Its method of establishing whether a property holds is to test for the refinement of a transition system capturing the property by the candidate machine. It also has the ability to check the determinism of a state machine, and this is used primarily for checking security properties [34].

Since using FDR2 modeling language is very time consuming and error prone [32], Casper [35] has been used, which is a compiler for analyzing security protocols. This combination is also known as the Casper/FDR2 approach and has been used in many works [32,34,36,37]. A Casper input file consists of eight headers that are described in Table 6. Using this input file, Casper will be able to generate a CSP compatible syntax, which will be then checked by FDR2.

#### 8.1.2. Security Proof

ECDH and HMAC are added into the design, and the rest of the security in Seluge++ depends on the hash dependency pattern of Seluge. Therefore, our claim of an authentic protocol depends on the security proof of new features. Consequently, to make sure that the generated key is only shared between the base station and the node, the key generation of Seluge++ is implemented in Casper with the help of the given examples in Casper's installation, as shown in Algorithm 4.



**Algorithm 4** Seluge++ key generation implemented in Casper.
1:#Free variables2:datatype F = G | Exp(F,Num) unwinding 23:BS, ND: Agent4:Ds, Dn: Num5:N1: Nonce6:Pub: Agent – > PublicKey7:Pri: Agent – > SecretKey8:H: HashFunction9:pwd: Agent x Agent – > Password10:InverseKeys = (sk,sk), (Exp, Exp), (Pub, Pri),(pwd, pwd)11:sk, Yn,Ys: F12:#Processes13:BASE(BS, Ds, SK) knows Pub, Pri(BS), pwd(BS,ND)14:MOTE(ND,Dn, N1, SK) knows Pub, pwd(BS,ND)15:#Protocol description16:0. – > BS : ND17:[BS ! = ND]18:1. BS – > ND : {Exp(G, Ds)%Qs}{Pri(BS)}19:[BS ! = ND and isExp(Qs)]20:< sk := Exp(Qs, Dn) >21:2. ND – > BS : Exp(G, Dn)%Qn, {Exp(G, Dn)%Qn}{pwd(BS, ND)}22:[ND ! = BS and isExp(Qn)]23:< sk := Exp(Qn, Ds) >24:3. BS – > ND : BS25:4. ND – > : sk26:#Equivalences27:forall x,y : Num . \28:Exp(Exp(G,y), x) = Exp(Exp(G,x), y)29:#Specification30:– –**Passed this test**31:Secret(BS, sk, [ND])32:– –**Passed this test**33:Secret(ND, sk, [BS])34:– –**Passed this test**35:WeakAgreement(BS,ND)36:#Functions37:inline isExp(y) = member(y, F__(1)) and y! =G38:symbolic Pub, Pri, pwd39:#Actual variables40:BaseStation, Node, Attacker : Agent41:W, ds, dn: Num42:n1, n2: Nonce43:#System44:BASE(BaseStation, ds)45:MOTE(Node, dn, n1)46:#Intruder Information47:Intruder = Attacker48:IntruderKnowledge = {Node, Intruder, W, n2, Pri(Attacker)}


**Figure 8.**
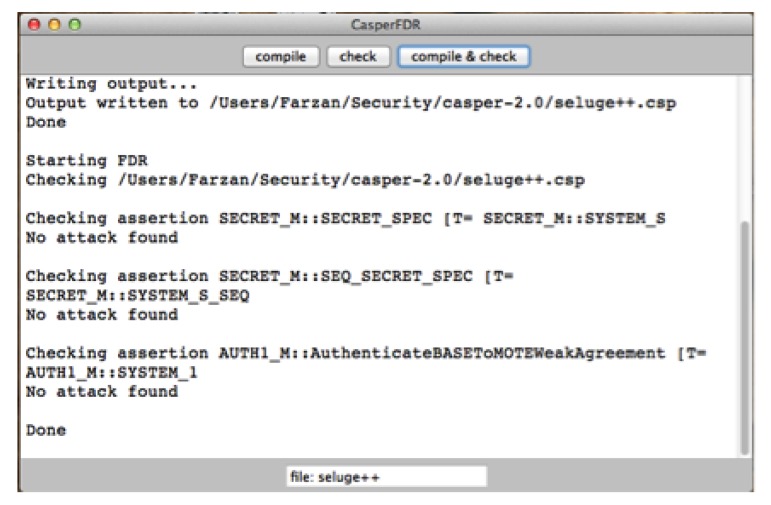
The FDR2 (Failures-Divergence Refinement) results of Seluge++ key generation in Casper.

ECDH key agreement is prone to man-in-the-middle attacks, unless it is used in an authenticated channel. As the communication medium of Seluge++ is not authenticated, Seluge++ is evaluated against this attack. The results of FDR2 (Figure 8) show that Seluge++ is able to successfully establish a shared session key, which is only accessible among the base station and the node. In Algorithm 4, after the session key (*SK*) is successfully established, another session key (pwd(BS, ND)) is used, because of the limitations in Casper. This does not violate any rule, because *SK* is tested to be secure, and the results in Figure 8 show this fact; also, pwd(BS, ND) is not included in the intruder's knowledge section, which means the attacker does not have a value of pwd(BS, ND).

### 8.2. Compatibility with SMR

As we have discussed in Section 5, there are a number of prerequisites that are essential to be employed by any OTAP scheme in order to make it secure. Consequently, Seluge++ complies with SMR to provide an improved secure scheme that not only preserves the security concepts (e.g., confidentiality, integrity, availability, authenticity), but also provides robustness against very common attacks that occur during the dissemination of a code update, like replay, battery-drain and wormhole attacks. Table 7 shows how Seluge++ complies with SMR.

Skipjack is a block cipher developed by the U.S. National Security Agency (NSA) [38]. It was first proposed as the encryption algorithm in a U.S. government-sponsored scheme of key escrow and used only for encryption. The design was initially secret, but was later declassified. Skipjack is a 64-bit cipher that utilizes an 80-bit crypto variable. Some security analysis has been done by researchers to show Skipjack's strength and to highlight the advantage of using it in wireless sensor networks [38–42].

Another feature used in Seluge++ to preserve the integrity and authentication is the hash function of SHA-1 and the keyed message authentication code (HMAC) based on SHA-1. Researchers have published some works on the security analysis of SHA-1 [43,44] and the security analysis of HMAC [45,46]. These works can be used to determine the suitability of these algorithms in wireless sensor networks.

As noted in SMR, a combination of immediate packet verification and HMAC prevents any successful attack against Seluge++. Since each packet is immediately verified as soon as it arrives at the node end, no successful DoS attack is possible. In Seluge++, Line 44 through 46 of Algorithm 2 is responsible for preventing DoS attacks. A symmetric key is also vulnerable to brute-force attacks. Since the symmetric key in Seluge++ is randomly generated for each entire process, therefore it is very unlikely to encounter a successful brute-force attack.

To solve the authentication delay problem, SMR suggests using message authentication code. When MAC is used, packets can arrive out of order, and the node can verify them without the need to wait for previously ordered packets. In Seluge++, this is helpful while sending page 0, which contains the hash values of page 1, and therefore, all other pages can be immediately verified as soon as they arrive at the node level, resulting in no authentication delay problems.

### 8.3. Performance Evaluation

TOSSIM is an event-based simulator, which operates on this TinyOS [47] platform. This can be used to simulate radio communications, as well as protocol implementations. There is an extension provided to it named PowerTOSSIM [48], which is used to simulate the power consumption of TinyOS applications. In TOSSIM, the same as TinyOS, the programming language is nesC [49], a dialect of the C language, which is optimized for resource constraint networks, like WSNs. TOSSIM and PowerTOSSIM have been used to evaluate Seluge++ for some energy consumption and also radio communications in terms of the time taken to disseminate all the update data.

ATMEL Studio is a development tool with a built-in simulator provided by the ATMEL Company for its AVR microcontrollers. Since some WSN nodes, like Mica2, use ATMEL products (ATMEGA128L), therefore for the calculation of the CPU clocks of the security primitives, ATMEL Studio is used. Mica2 is the target of our simulations, and some useful information from the Mica2 datasheet [50] is provided in Table 8.

#### 8.3.1. SkipJack

The SkipJack algorithm has been used to provide confidentiality in the Seluge++ design. Its performance has been tested to show its execution time in a network. ATMEL Studio is used to test eight bytes of data on an ATMega128 microcontroller. Since ATMega128 is used, the results of this simulation are the same as if the source code were run on a Mica2 mote. The implementation of SkipJack is tested on a non-optimized assembly code. Table 9 describes the results of this simulation.

Based on the results provided in Table 9, the encryption of a single 64-bit block with an 80-bit key in a mote running a 16 Mhz CPU clock will take approximately 3.6 *μ*S, which gives us a reasonable energy consumption throughout the complete dissemination of the code update using the following calculation (core voltage = 3.0 V, time = 3.6 *μS*, current = 8 mA (extracted from Table 8)):

$$\begin{array}{c} J=V\times C(\text{Coulomb})=V\times (I\times t)=\\ 3.0(V)\times (8\times 10^{-3})\times (3.6\times 10^{-6})=86.4(\mu J) \end{array}$$

#### 8.3.2. SHA-1

The results of the simulation of 160-bit SHA-1 algorithm implementation in the assembly language and tested in ATMEL Studio are shown in Table 10. Therefore, the energy consumption of this algorithm can be calculated as below:

$$\begin{array}{c} t=\frac{29,193}{16,000,000}=1.82(ms)\\ J=3.0(V)\times (8\times 10^{-3})\times (1.82\times 10^{-3})=43.68(\mu J) \end{array}$$

### 8.4. Overhead Improvement and Stronger Security

We use the term stronger security, because hash algorithms with any block length can be used in Seluge++, and the security of Seluge++ will highly depend on the used hash algorithm. Seluge++ has replaced the Merkle hash tree with a new mechanism described in Section 6.4. The following analysis is done to show that the replaced mechanism is better, both in the initial overhead and the maximum provided block length support for the hash algorithm.

The usage of a Merkle hash tree incurs some overhead on the number of packets to be disseminated over the network. Because of the reminding behavior of the Merkle hash tree and its relative Equation (1) in Section 2 and also because the maximum authentication path (AP) length in a Merkle Tree is equal to *l* in 2*^l^* [51], it can be identified that each hash packet needs to be sent with *K* more hash values located in its AP. Therefore, to send all the hash packets in page 0, which are identified using *M*, it is necessary to send a number of *H* hash values equal to the result of Equation (5) below:

(5) H=K×M

To find out how many overhead packets (*O*_1_) are implied in their mechanism, it is required to multiply the value of H by the number of bytes used by the hash algorithm (|*H*(.)|) and divide the results by the payload size of each packet (*B*). Therefore, the total implied overhead packets will be calculated using Equation (6):

(6) $$O_{1}=\frac{K\times M\times |H(.)|}{B}$$

Since (*M* = 2*^K^*), Equation (6) can be rewritten as below:

(7) $$O_{1}=\frac{K\times 2^{K}\times |H(.)|}{B}$$

On the other hand, the mechanism employed by Seluge++ first merges all the hash values of page 1 to create the packets of page 0. The number of packets in page 0 (*NP*0) in absence of the required bytes for MAC can be calculated using Equation (8):

(8) $$NP0=\left\lceil \frac{\text{NPPP}\times |H(.)|}{B}\right\rceil$$

Each packet in page 0 needs to be merged with a number of MAC bytes for authentication and integrity check purposes. The number of packets in page 0 in the presence of the required bytes for MAC (denoted as *I*) can be calculated using Equation (2) in Section 6.3. This will create the initial overhead of Seluge++ and can be calculated using Equation (9):

(9) $$O_{2}=I-NP0=\left\lceil \frac{\text{NPPP}\times |H(.)|}{B-|{\text{MAC}}_{SK}(.)|}\right\rceil -NP0$$

Having Equations (7) and (9), it can be easily identified that Equation (9) has a great advantage over Equation (5), because it is a linear equation, while in the other side Equation (5), it has an exponential increase with am increment value of *K*.

**Figure 9.**
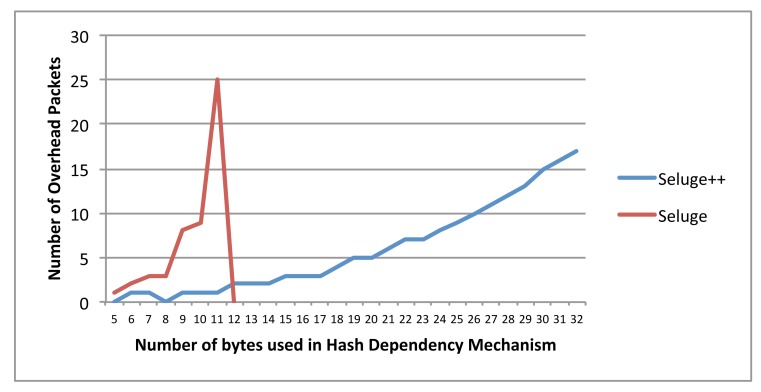
Overhead packets using different hash block lengths (from 40-bit to 256-bit).

**Figure 10.**
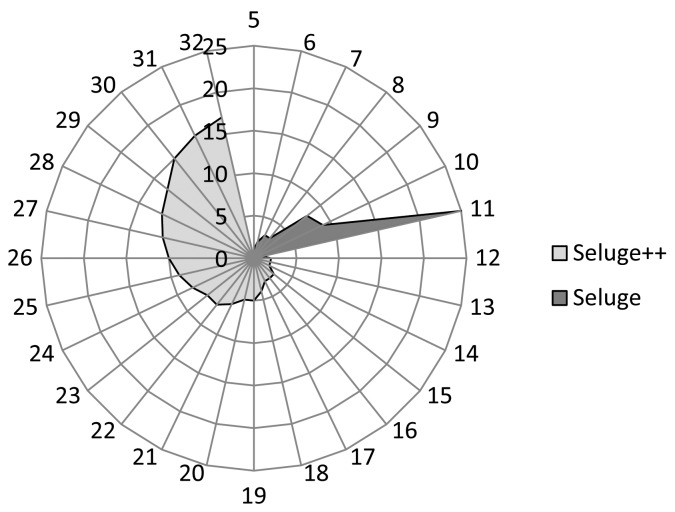
Range support of Seluge and Seluge++ for a range of sequential hash block lengths.

Considering the above facts, two major differences will be identified between Seluge and Seluge++. In the case of Seluge, the increase in the hash strengthening will have an exponential increase in the number of overhead packets (*O*_1_), while in Seluge++, it will be a linear increment (Figure 9). Furthermore, as discussed in Section 3, an analysis on the original Seluge shows that it does not support hash values having more than 11 bytes in their output. Seluge++ can support all possible hash bytes with minimum possible overhead even compared with the best case of the original Seluge (Figure 10). For the original Seluge, in the best case with the highest possible hash bytes (|*H*(.)| = 11) and having the default Seluge settings (*NPPP* = 48, *B* = 72), it requires 25 overhead packets, while in the same situation, Seluge++ has an overhead of only one packet.

In Figure 9, an analysis on both Seluge++ and the original Seluge using different hash block lengths has shown that Seluge++ needs a very lower number of overhead packets for its initial integrity and authentication check compared to the original Seluge, which has an exponential increase in the number of required packets. The disappearance of Seluge's line in this diagram shows no support for the larger block length.

Figure 10 illustrates the fact that Seluge++ has full coverage of all hash algorithms with any block length, but the original Seluge support ends in a hash algorithm with a maximum block length of 11 bytes. The original Seluge has a 25 packet overhead for only a hash block length with 11 bytes, while for the same value, Seluge++ has only one byte of overhead.

### 8.5. Overall Energy Consumption

As previously mentioned, the main difference between Seluge++ with Seluge is the process of sending hash values in page 0. We have analyzed this difference in different ways (e.g., security, performance). To compare the energy consumption caused by changes made on Seluge's design, the energy consumption of each component is calculated, and, then, an overall comparison is presented.

Every complete propagation of the code update in both schemes consists of two phases: *Phase 1* is the initialization process, which includes sending the hash packets of page 0; and *Phase 2* is sending image data (data transmission). In Seluge, the overall energy cost of Phase 1 (Seluge-init) can be calculated by measuring the energy required to create the Merkle tree and sending its packets over a network. Note that the energy required for signing an authentication packet is not considered in this phase, because each scheme can choose to use different authentication mechanisms. For each node in the Merkle tree, Seluge needs to execute a hash function. Therefore, the energy required in this part is (2*^k^* − 1 + 2*^k^*) × *E*(*SHA*1). The energy for sending all packets caused by the Merkle tree is equal to (*O*1 + *NP*0) × *B* × *E*(*Tx*). For Phase 2 in Seluge (Seluge-data), we can use *E*(*SHA1*) × (*ImageSize*/(*B* − |*SHA*1|)) to calculate the energy required for sending all of the image data, including their hash dependency values and the energy required for creating the hash dependency. On the other side, in Phase 1 of Seluge++ (Seluge++-init), the overall energy consumption is calculated by measuring the energy required for executing the HMAC function for every packet in page 0 and transmitting these packets (*I* × *E*(*HMAC*)) + (*I* × *B* × *E*(*Tx*)). Additionally, an ECDH mechanism will need a specific amount of energy that has to be considered *E*(*ECDH*). The energy calculation of Phase 2 in Seluge++ (Seluge++-data) is almost the same as Seluge, but having an additional value of encrypting the whole image data (*ImageSize*/8 × *E*(*SkipJack*)). In summary, for computing the overall energy required for each of these schemes, the equations can be written as shown in Table 11.

Since HMAC specification in RFC-2104 shows that computing HMAC over some *data* using a secret key (*K*) is defined like *H*(*K* ⊕ *opad, H*(*K* ⊕ *ipad, data*)), therefore the approximate energy required for HMAC execution can be denoted as two times the energy required for its underlying hash function. As we use the SHA-1 hash function in Seluge++, therefore the energy required for HMAC (*E*(*HMAC*))) is considered to be equal to 2 × 43.68 *μJ* = 87.36 *μJ*. Furthermore, since we do not have the energy required by SHA-1 with block lengths other than 160-bit, the results provided in Section 8.3.2. are used in our experiment. The TinyECC library is considered to provide ECDH key exchange functionality in Seluge++. Experimental evaluations published for TinyECC shows that the ECDH key established operation needs 50.82 mJ for the MicaZ platform [29]. Since MicaZ is the closest platform to Mica2, therefore 50.82 mJ is used for the energy evaluations of ECDH in this paper. The energy cost of transmitting one byte (*E*(*Tx*)) is also considered to be 59.2 *μJ* [21].

**Figure 11.**
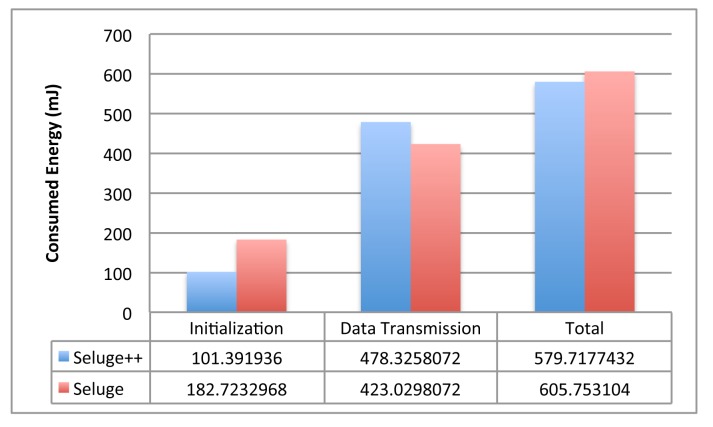
The energy comparison of Seluge and Seluge++ with an 11-byte hash block length.

**Figure 12.**
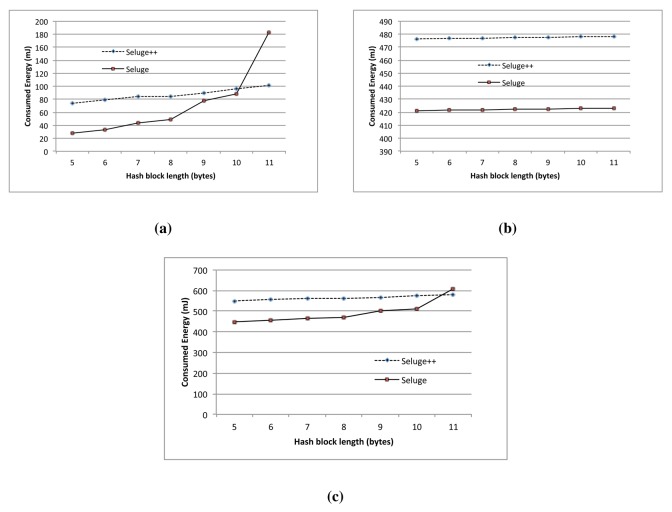
Comparison of the overall energy cost in the dissemination of 5-kb image data. (**a**) Initialization phase; (**b**) Data transmission phase; (**c**) Total.

Figure 11 shows that with an 88-bit block length of the hash function, the initialization phase of Seluge++ has less energy consumption than Seluge, while the data transmission phase of Seluge++ has more energy cost. Seluge's advantage in data the transmission phase is typically because of the energy consumed for the encryption employed in Seluge++. Figure 12 illustrates the overall advantage of Seluge's energy consumption over Seluge++'s energy cost when two protocols are run in the same conditions. However, considering the improved security provided by Seluge++, we believe that this extra cost is acceptable. Figure 12 also shows that the energy consumption growth of Seluge is exponential, and with hash block lengths equal and larger than 11 bytes, Seluge++ has the advantage over original Seluge in terms of energy cost. Consequently, it is suggested to use Seluge++ in situations where security must be very restricted, and therefore, hash functions with block lengths larger than 11 bytes are used. On the other hand, Seluge is better for regular situations where smaller block lengths are acceptable. As described in Section 8.4, Seluge does not support hash block sizes larger than 11 bytes, but Seluge++ does. Moreover, Seluge++'s overhead is higher than Seluge only when using hash block sizes smaller than 11 bytes (Figure 12c). In this range, none of these two schemes can provide a reasonable security level for mission-critical situations. When 11 bytes is selected for the hash block size used in both schemes, Seluge++'s overhead is better than the overhead that Seluge incurs (Figure 11). Consequently, If we use large hash block sizes to provide a greater security level, which is the purpose of proposing Seluge++, then the overhead incurred would be less than Seluge's overhead.

In other word, Seluge++ has an improved overhead rate compared to what Seluge provides with its less secure scheme. This improvement is effective on hash block sizes equal and larger than 11 bytes (Shown in Figure 12c).

However, we believe that since Seluge++ is proposed for hostile environments, it is better to use it with block sizes equal to and larger than 11 bytes, because below this range, it has a higher overhead compared to Seluge. Seluge still remains the best scheme to be used in standard environments in which security need not to be very strict only, and only in terms of overhead. However, when it comes to security, Seluge++ still is better than Seluge, even while using hash block sizes smaller than 11 bytes. Albeit that Seluge++ will have more overhead when being used in this range of hash block sizes, it provides a more secure scheme; therefore, if this overhead is tolerable in-field, then it is suggested to use Seluge++ even in non-hostile environments.

## 9. Conclusion

Existing security models used in over-the-air dissemination of code updates in WSNs perform well in applications where the security requirement needs to be at its standard level, but suffers in applications where it needs to be at its maximum level. Considering this, we searched for possible vulnerabilities in existing security solutions, and then, we provided a set of countermeasures correspondingly named Security Model Requirements (SMR). Based on the investigation, we proposed an improved version of Seluge named Seluge++. Similar to Seluge and other existing OTAP protocols, Seluge++ relies on the Deluge protocol for code dissemination. It complies with SMR and fulfills all these security requirements. We have provided a detailed description for each implemented element to show that Seluge++ preserves confidentiality, provides a suggestion of how to preserve data privacy, mitigates attacks that threaten the availability property of this secure model (e.g.,DoS attack) and also successfully mitigates replay, battery-drain and wormhole attacks against dissemination of the code update. Security and performance analyses have shown that Seluge++ has a great advantage over other mechanisms in terms of maintaining a balance between energy consumption and providing the highest possible level of security.

In testing the performance of Seluge++, we have used simulation and numerical analysis. The performance study of it in a real WSN test-bed is a recommended future research direction. In the experiment, we considered only Mica2 sensor nodes, which is a popular sensor node. Investigations of other nodes would be of merit.

## Figures and Tables

**Table 1.** A summary of the existing security models for the the over-the-air programming (OTAP) schemes.

**Name**	**Privacy/Confidentiality**	**Authentication**	**Integrity**	**DoS Resilience Attack Resilience**	**Wormhole Attack Resilience**	**Replay Attack Resilience**	**Battery-Drain**	**Overhead**	**Comments**
Deluge [23]	×	×	×	×	×	×	×	×	Efficient Reprogramming Scheme
Sluice [11]	×	✓	✓	×	×	×	×	L	-
*μ*PKI [21]	✓	✓	✓	×	×	×	×	VH	-
Chain-Based [22]	×	✓	✓	×	×	×	×	L	Ordered Delivery of Packets
Hash-Tree [22]	×	✓	✓	×	×	×	×	VH	-
Hybrid [22]	×	✓	✓	NVG	×	×	×	VH	-
Seluge [12]	×	✓	✓	A	✓	✓	×	VH	Limited Support for Hash Algorithms
Das and Joshi Scheme [20]	×	✓	✓	×	×	×	×	L	Impersonation Attack [24]

A, acceptable; NVG, not very good; VH, very high; L, low; ×, not considered in the design; ✓, full preservation.

**Table 2.** Security Model Requirements (SMR).

**Security Principle**	**Requirement**
Confidentiality	An encryption algorithm; symmetric is preferred.
Authentication	Asymmetric Cryptography (Signature Verification).
Integrity	Hash dependency of data combined with PKC signature verification; and/or message authentication code
Availability (DoS Attack Resilience)	Immediate verification at the packet-level.
Replay Attack Resilience (freshness must be preserved)	Inserting a constant and unique number for each version to be comparable for each node to detect repeated data.
Worm-Hole Attack Resilience	Avoid using the same symmetric cryptography keys in the whole network.
Battery-Drain Attack Resilience	Using weak authentication on packets that have expensive computations on the time of reception; and, an auto-halting system that will partially protect the system from resource exhaustion.

**Table 3.** The energy cost of cryptographic primitives (millijoules).

**Algorithm**	**Signature**		

	**Sign**	**Verify**
RSA-1024	304	11.9

ECDSA-160	22.82	45.09

RSA-2048	2302.7	53.7

ECDSA-244	61.54	121.98

**Table 4.** Notations. ECC, elliptic curve cryptography; MAC, message authentication code.

**Name**	**Symbol**	**Description**
Global Public Key of Server	*GK_pub_*	TinyECC Public Cryptography library will be used.
Global Private Key of Server	*GK_Pri_*	TinyECC Public Cryptography library will be used.
Asymmetric Signature	*Sign_key_*(*X*)	Sign X with a key using an asymmetric algorithm (TinyECC).
Signature Verification	*Verify_SK_*(*X*)	Denotes signature verification of value X with a key using an asymmetric algorithm (TinyECC).
Ceiling	⌈*x*⌉	Gives the smallest integer greater than or equal to *x*.
Elliptic Curve Domain parameter	*T*	*T* = (*p, a, b, G, n, h*).
ECDH Server Secret Key	*d_S_*	A random number in the range of [1, *n* − 1].
ECDH Node Secret Key	*d_N_*	A random number in the range of [1, *n* − 1].
ECDH Public Key of Server	*Q_S_*	*d_S_G*
ECDH Public Key of Node	*Q_N_*	*d_N_G*
Session Key	*SK*	*d_S_Q_N_ OR d_N_Q_S_*. It is the session key as introduced in Section 5.
Byte Numbers of Shared Key	*Z*	Default is 24 = 192-bit, but for SkipJack, only *Z* = 10 bytes will be used.
Number of Hash Packets	*I*	Number of hash values in page 0 after MAC authentication is added to them †.
Version Number	*V_n_*	Incremental number per each new version.
Nonce	*N*_0_ … *N_n_*	A random 160-bit number.
Number of Packets Per Page	*NPPP*	Default is 48.
SHA-1 Hash Bits	*r*	For example, *r* = 160 means SHA-1 160 bit.
Number of Bytes Per Packet	*B*	Payload size of a packet (default is 92, maximum is 102).
One-way Hash Function	*H^l^*(*X*)*orH^l^*(.)	Denotes the output of running a l-bit one-way hash function (H) on value X.
Message-Specific Puzzle	$H_{n}^{l}(V)$	An l-bit one-way hash function of H on the value of V, which is repeated n times; $H_{1}^{64}(V)=H^{64}(V)H_{2}^{64}=H^{64}(H_{1}^{64}(V))$ and so on.
		This method is called message-specific puzzles and fully described in [16].
Size	|*X*|	Denotes a size of *X* in bytes.
Symmetric Encryption	${\text{Enc}}_{sk}^{l}(X)$	Symmetric encryption of X using an l-bit session key (SK).
Symmetric Decryption	${\text{Dec}}_{sk}^{l}(X)$	Symmetric decryption of X using an l-bit session key (SK).
Message Authentication Code	${\text{MAC}}_{sk}^{l}(X)$ or ${\text{MAC}}_{sk}^{l}(.)$	Denotes the generation of a message authentication code out of X with an l-bit shared key (SK) by using a keyed one-way hash function.
Packet Data	{A,B,C}	Means a packet with data variables of A, B and C.
Packet Pointer	Pkt[n]	n-*th* variable of a packet (*n* ≥ 0).
Energy	*E*(*x*)	Denotes the energy required for an operation *x* to be completed on a mote in joules.

† This can be calculated using Equation (2):

**Table 5.** Seluge and Seluge++ comparison in terms of security support. DoS, denial of service.

**Name**	**Privacy/Confidentiality**	**Authentication**	**Integrity**	**DoS Resilience**	**Wormhole Attack Resilience**	**Replay attack Resilience**	**Battery-Drain Attack Resilience**	**Overhead**	**Maximum hash Block Size**	**Comments**
Seluge	×	✓	✓	A	✓	✓	X	VH	11	Limited Support for Hash Algorithms

Seluge++	✓	✓	✓	✓	✓	✓	✓	VL	∞	-

A, acceptable; VH, very high; VL, very low; ×, not considered in the design; ✓, full preservation.

**Table 6.** Header description of Casper's input file.

**Header**	**Description**
#Free Variables	Defines the agents, custom types and variables.

#Processes	Is actually the list of agents.

#Protocol Description	Shows the messages that has to be exchanged between agents. This is actually the implementation of the protocol.

#Specification	Security properties that have to be checked (e.g., the secrecy of a session key).

#Actual Variables	Real variables. These will help to specify which of the free variables have to be renewed per each run of the protocol for each agent.

#Function	User-custom functions can be defined here.

#System	Instantiates agents with real variables.

#Intruder Information	The attacker capabilities must be specified here.

**Table 7.** Security features of Seluge++. CBC, cipher-block chaining.

**Security Principle**	**Technique Used in Seluge++**
Confidentiality	SkipJack block cipher with CBC mode is chosen

Authentication	TinyECC library in which an ECC library is highly adapted for WSN is used

Integrity	Hash dependency technique of the original Seluge is used for data packets, and for hash packets in page 0, a message authentication code is used

Availability (DoS Attack Resilience) and Authentication Delay Problem	Immediate verification provided by hash dependency in data packets and MAC verification in hash packets together with the message-specific puzzle feature, which can be disabled and then re-enabled during an attack, which will mitigate DoS attacks against Seluge++

Replay Attack Resilience	Inserting a constant and unique number for each version to be comparable for each node to detect repeated data (version number *V_n_* defined in Section 6.4)

Worm-Hole Attack Resilience	A temporary and unique session key per each communication link will protect Seluge++ from Wormhole attacks against dissemination of the code update

Battery-Drain Attack Resilience	Using weak authentication on packets that have expensive computations on the time of reception (e.g., Message-Specific Puzzle); an auto-halting system that will partially protect the system from resource exhaustion

**Table 8.** Mica2 mote specification [50].

**Function**	**Corresponding Value**
Input Voltage	2.7–3.3 V

Current Draw	8 mA

Configuration EEPROM	4 Kbytes

Transmission Current Draw	27 mA

**Table 9.** Performance analysis of SkipJack.

Program Memory Usage	1,464 bytes	

Data Memory Usage	27 bytes	

CPU Cycles	Encryption	5780

	Decryption	5736

**Table 10.** Performance analysis of SHA-1.

**Platform**	**CPU Clocks**	**Read-Only Memory (ROM) Size (Bytes)**
Assembly in ATMEL Studio	29,193	4,286 bytes

**Table 11.** The formulas used for the energy cost calculation of Seluge and Seluge++.

**Protocol-Phase**	**Formula**
Seluge-init	((2*^K^* − 1 + 2*^K^*) × *E*(*SHA*1)) + ((*O*_1_ + *NP*0) × *B* × *E*(*Tx*))
Seluge-data	$\left(E(\text{SHA}1)\times \left\lceil \frac{\text{ImageSize}}{B-\left|\text{SHA}1\right|}\right\rceil )+(E(Tx)\times B\times \left\lceil \frac{\text{ImageSize}}{B-\left|\text{SHA}1\right|}\right\rceil \right)$
Seluge++-init	(*I* × *E*(*HMAC*)) + (*I* × *E*(*Tx*) × *B*) + *E*(*ECDH*)
Seluge++-data	$\text{Seluge}‐\text{data}+(\frac{\text{ImageSize}}{8}\times E(\text{SkipJack}))$
